# Sampling Device-Dependence of Prokaryotic Community Structure on Marine Particles: Higher Diversity Recovered by *in situ* Pumps Than by Oceanographic Bottles

**DOI:** 10.3389/fmicb.2020.01645

**Published:** 2020-07-15

**Authors:** Viena Puigcorbé, Clara Ruiz-González, Pere Masqué, Josep M. Gasol

**Affiliations:** ^1^School of Science, Centre for Marine Ecosystems Research, Edith Cowan University, Joondalup, WA, Australia; ^2^Department of Marine Biology and Oceanography, Institut de Ciències del Mar (ICM-CSIC), Barcelona, Spain; ^3^Institut de Ciència i Tecnologia Ambientals (ICTA), Bellaterra, Spain; ^4^Department of Physics, Autonomous University of Barcelona, Barcelona, Spain; ^5^International Atomic Energy Agency, Monaco City, Monaco

**Keywords:** prokaryotic communities, marine particles, size-fractionation, *in situ* pumps, oceanographic bottles, polynyas

## Abstract

Microbes associated with sinking marine particles play key roles in carbon sequestration in the ocean. The sampling of particle-attached microorganisms is often done with sediment traps or by filtration of water collected with oceanographic bottles, both involving a certain time lapse between collection and processing of samples that may result in changes in particle-attached microbial communities. Conversely, *in situ* water filtration through submersible pumps allows a faster storage of sampled particles, but it has rarely been used to study the associated microbial communities and has never been compared to other particle-sampling methods in terms of the recovery of particle microbial diversity. Here we compared the prokaryotic communities attached to small (1–53 μm) and large (>53 μm) particles collected from the mesopelagic zone (100–300 m) of two Antarctic polynyas using *in situ* pumps (ISP) and oceanographic bottles (BTL). Each sampling method retrieved largely different particle-attached communities, suggesting that they capture different kinds of particles. These device-driven differences were greater for large particles than for small particles. Overall, the ISP recovered 1.5- to 3-fold more particle-attached bacterial taxa than the BTL, and different taxonomic groups were preferentially recovered by each method. In particular, typical particle-attached groups such as Planctomycetes and Deltaproteobacteria recovered with ISP were nearly absent from BTL samples. Our results suggest that the method used to sample marine particles has a strong influence in our view of their associated microbial communities.

## Introduction

The biological carbon pump (Volk and Hoffert, 1985) is a key component of the global carbon cycle, removing CO_2_ from the atmosphere by gravitational settling of particulate organic carbon. Microbial decomposition of particles during sinking affects their properties, in general leading to the attenuation of the flux of carbon with depth (Herndl and Reinthaler, 2013; Legendre et al., 2015). In fact, only a small percentage (<5%) (Antia et al., 2001; Honjo et al., 2008) of the particulate organic carbon produced in surface waters reaches abyssal depths, and whether the decomposition processes occur in upper or in deep ocean layers determines how long carbon is sequestered in the ocean (Kwon et al., 2009). Since not all microbes affect organic matter in the same manner (Kujawinski et al., 2016; Robinson et al., 2018), the structure of the microbial community growing on the sinking particles will impact the efficiency of carbon sequestration.

A number of studies have characterized the microbial communities colonizing the material that potentially sinks to the deep ocean, unveiling that the prokaryotes attached to those particles differ from free-living assemblages (DeLong et al., 1993; Rieck et al., 2015) and between different particle types (Mestre et al., 2017; Duret et al., 2019), display compositional changes during sinking (Pelve et al., 2017), and might even source prokaryotes to the bathypelagic realm (Mestre et al., 2018). In general, most studies on deep-sea marine particles have used size-fractionation to capture the microbes associated with both suspended and sinking material, collecting seawater with Niskin oceanographic bottles and filtering it through different pore-size filters. Other less used methods include sediment traps, which recover mainly sinking particles but might miss those with low sinking rates (i.e., the so-called suspended particles; LeCleir et al., 2014; Fontanez et al., 2015; Boeuf et al., 2019), or marine snow-catchers, which allow separating the suspended and sinking material present in a sample by their sinking velocities (Duret et al., 2019). All these methods involve a certain time lapse between collection of particles and storage of samples (ranging from several hours in the case of sampling with oceanographic bottles to several days, or even weeks, in sediment traps) that might result in changes in particle-attached microbial communities. Even in those cases where a fixative is added to sediment traps (e.g., Gutierrez-Rodriguez et al., 2019), it is possible that not all microbial groups are well preserved if the trap is deployed at depth for a long time or that the particle-microbiome structure is not well preserved.

*In situ* size-fractionated filtration with submersible pumps (ISP) is widely used in marine biogeochemical studies for collecting suspended and sinking particles down to 5000 m (McDonnell et al., 2015, GEOTRACES program^1^). Besides reducing the time between particle collection and storage, the *in situ* filtration keeps the sampled microbial communities at *in situ* conditions for most of the time, thus minimizing the growth of typical copiotrophic bacteria upon changes in pressure and temperature. There is an increasing interest in developing sampling systems based on *in situ* filtration (e.g., the Clio device, Jakuba et al., 2018, or the ISMFF—*in situ* microbial filtration and fixation, Wang et al., 2019) to facilitate large-scale microbial ecology studies (e.g., Saito et al., 2019). So far, however, ISP have not been extensively used to sample particle-attached microbial communities (Lincoln et al., 2014), and thus, it is not known whether they successfully capture particle-attached microbial diversity in comparison to other more commonly used strategies.

Several studies comparing the chemical nature of particles (particularly organic carbon, nitrogen, and trace metals) collected with different methods have reported chemically different types of particles recovered even when the type and pore size of filters are the same (Gardner et al., 2003; Liu et al., 2005, 2009; Bishop et al., 2012; Puigcorbé et al., 2015; Twining et al., 2015). Recent studies have attempted to compare whether the recovered microbial communities differ when sampling with oceanographic bottles or with *in situ* filtration systems (Torres-Beltrán et al., 2019; Wang et al., 2019) reporting device-driven taxonomic differences in most cases, but these studies targeted mostly free-living communities. To the best of our knowledge, no study has explicitly assessed the impact that the sampling device can have on the recovered structure of the prokaryotic communities associated with marine particles.

In this context, we explored whether sampling marine particles with oceanographic bottles (BTL) or *in situ* pumps (ISP) has an impact on our view of the structure of the associated microbial communities. Using Illumina sequencing of the 16S rRNA gene, we examined the method-driven differences on the prokaryotic communities associated with small (1–53 μm) and large (>53 μm) particles collected at mesopelagic depths from two Antarctic polynyas. Large particles (>50 μm) have historically been considered to be the main vectors for carbon (and other elements) export from surface waters to depth, although there is a growing body of literature highlighting the importance of smaller particles (∼1–50 μm) in the marine biogeochemical cycles (Durkin et al., 2015; Puigcorbé et al., 2015; Le Gland et al., 2019). Knowledge on the effect of the sampling device on the recovery of microbial communities associated with particles of different sizes is essential for a deeper understanding of particle microbial ecology, and ultimately, their role in defining the efficiency of the transport of carbon and other elements of interest to depth.

## Materials and Methods

### Study Area

Sampling was performed on board of the R/V Aurora Australis (AA-V02 2016/17) between December 8, 2016, and January 21, 2017, in the Dalton and the Mertz polynyas (East Antarctica; 67.2–66.8°S and 119.5–145.8°E, Table 1). Polynyas are highly productive, with average phytoplankton concentrations being more than twice of all the open waters around Antarctica (Arrigo et al., 2015). Polynyas are areas where particle fluxes are expected to be significant, and they also harbor high densities of upper trophic level organisms such as birds and marine mammals (Karnovsky et al., 2007). Further information regarding the sampling area can be found in Moreau et al. (2019). Samples were collected at two mesopelagic depths (100 and 300 m), using either a 12L-Niskin bottle (BTL-samples) or an *in situ* pump (ISP-samples) deployed at each depth.

**TABLE 1:** Station ID, sampling date, coordinates, depth and hydrochemical variables.

Polynya	Station	Date	Lat. (°N)	Lon. (°E)	Depth (m)	Temp. (°C)	Sal.
Dalton	2	31/December/16	−66.8	119.5	100	−1.94	34.26
					300	−1.85	34.32
Mertz	EM03	10/January/17	−66.9	145.5	100	−1.57	34.45
					300	−1.86	34.56
	MG08	11/January/17	−67.2	145.9	100	−1.77	34.43
					300	−1.88	34.53

### Particle Sampling

For BTL samples, 7.5–10.5 L of water was collected in a carboy and filtered onboard sequentially through a 47 mm diameter 53 μm pore-size Nitex screen, followed by a 0.8 μm pore-size polycarbonate membrane filter (Millipore) (for simplification hereafter the 0.8–53 μm will be refered as 1–53 μm) using a Masterflex peristaltic pump. For ISP samples, the pumps (McLane) had heads (trace metal clean 3-tier model) equipped with a 142 mm diameter 53 μm pore Nitex screen, followed by a 1 μm pore quartz filter (QMA), and filtered, on average, 375 L of water during ∼2 h. To make the comparison of sampled volumes more even between the BTL and ISP, we took subsamples of the material retained in the ISP filters. The subsamples for the 1–53 μm faction were obtained by cutting 25 mm replicate punches from a 142 mm QMA filter. The >53 μm fraction was subsampled by rinsing the screen with filtered (<0.2 μm) seawater and dividing the obtained volume into various aliquots that were subsequently filtered through QMA filters. As a result, the 1–53 μm and >53 μm ISP samples were representative of about 10 and 35 L, respectively. Considering the volume filtered and the filter diameter for BTL and ISP, the ISP filtered on average about 4 times more volume per unit of area than the BTL (2 L cm^–2^ vs. 0.5 L cm^–2^).

Duplicates or triplicates were obtained from the 1–53 μm fractions and for most of the >53 μm fractions (Supplementary Figure S1). After the recovery of the ISP, the filters were prepared and stored at −80°C within the following hour. The processing time for BTL samples was about 2–3 times longer, with a CTD deployment of about 2 h, followed by storage at 4°C for a few hours until filtration and final storage at −80°C.

### Characterization of Prokaryotic Communities

The prokaryotic community structure was determined by high-throughput Illumina sequencing of the 16S rRNA genes. A total of 27 samples were obtained, including the replicates, which were stored at −80°C until further analyses. At the home laboratory, DNA was extracted using the PowerWater DNA Isolation kit following the manufacturer’s instructions (MOBIO). The samples were sequenced using Illumina MiSeq 2×300 bp flow cells at RTL Genomics (Texas, United States) using primers 515F-Y and 926R (Parada et al., 2015) to amplify the V4-V5 region of the 16S rRNA gene. The sequences were processed following (Logares, 2017). Quality-checked sequences were clustered into OTUs (operational taxonomic units) at 99% similarity. Singletons and chimeric OTUs were removed, and the remaining OTUs were taxonomically annotated using the SILVA v123 database. OTUs assigned to chloroplasts were removed, resulting in a total of 6,868 OTUs. To enable comparisons between samples, the OTU table was randomly subsampled to ensure an equal number of sequences per sample (5,000 sequences), retaining 133,885 sequences clustered into 5,786 OTUs. The raw sequence data have been deposited in the Figshare data repository, together with the non-rarefied OTU table, the taxonomy table and the environmental data used in this study, 10.6084/m9.figshare.12333107.

### Statistical Analyses

The spatial differences between prokaryotic communities were visualized using non-metric multidimensional scaling (NMDS, Vegan *metaMDS* function) based on Bray–Curtis distances. Significant differences in taxonomic composition between depths, stations, sampling method, or size fraction were tested using ANOSIM (Vegan *anosim* function). Device-driven differences in community structure for each size fraction were further calculated at each station as Bray–Curtis dissimilarities between ISP and BTL assemblages. OTUs unique to a given sampling method (i.e., OTUs present exclusively in BTL or in ISP) or shared between both methods were identified considering each sample separately. Finally, in order to explore whether each method recovered preferentially different taxonomic groups, indicator species analysis was done using the *indval* function of the R package LabDSV (Roberts, 2013), at a significance level of *p* ≤ 0.05, pooling all samples together. Statistical analyses and data handling were done in R (R Core Team, 2017).

## Results

**FIGURE 1:**
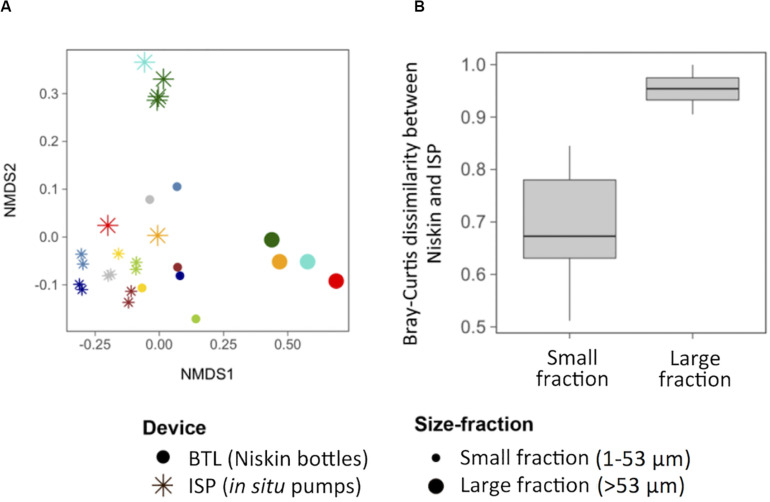
**(A)** Non-multidimensional scaling analysis (NMDS) based on the Bray–Curtis dissimilarity between all the studied communities collected with either *in situ* pumps (ISP, asterisks) or bottles (BTL, dots), for two different particle sizes (1–53 μm and >53 μm, indicated by the size of the symbol). Each color represents a community. **(B)** Box plot showing the distribution of pairwise Bray–Curtis dissimilarities between sampling devices for each size fraction.

The taxonomic composition of the studied particle-attached communities was first examined based on potential differences driven by station, depth, particle size, and/or sampling device. We observed that whereas communities did not cluster based on station or depth, particle size and sampling device resulted in clear taxonomic differences between communities (ANOSIM *R* = 0.54 and 0.43, respectively, *p* < 0.001, Figure 1A). The fact that the ISP replicates showed high similarities (Figure 1A) discards a bias due to subsampling of the ISP filter (see section “Materials and Methods”): Bray–Curtis dissimilarities between replicates of a sample were significantly smaller (0.301 ± 0.030) than between different samples (0.718 ± 0.007; Supplementary Figure S1). Notably, the device-driven differences in taxonomic composition of the microbial communities for a given sample were much more conspicuous for the large particles than for the small particles (Figure 1B), suggesting that the effect of the sampling method affects differentially particles of different sizes. Moreover, communities from the large size-fractions collected with ISP appeared more heterogeneous across sampling sites than BTL assemblages; whereas the former clustered into two groups, the latter clustered closely together (Figure 1A).

At the two stations in which both, large and small particles were collected (EM03 and MG08), we explored the Bray–Curtis dissimilarity between small and large fractions sampled with each device within a given site. We found that the average values were 0.51 ± 0.27 and 0.86 ± 0.11 for ISP and BTL, respectively (Table 2) indicating that the community structure in the two fractions were more similar in the ISP than in the BTL samples.

**TABLE 2:** Taxonomic differences (as measured by Bray–Curtis dissimilarity) between small and large size-fractions collected with either BTL or ISP devices.

Station	Depth	Device	Bray–Curtis dissimilarity (large vs. small)*
EM03	100	BTL	0.81
		ISP	0.46
	300	BTL	0.98
		ISP	0.26
MG08	100	BTL	0.73
		ISP	0.80
	300	BTL	0.93
		ISP	0.89

*Bray–Curtis values represent single values if only one sample replicate of each size fraction was available, or average of the pairwise distances in those cases where more than one replicate was collected.

**FIGURE 2:**
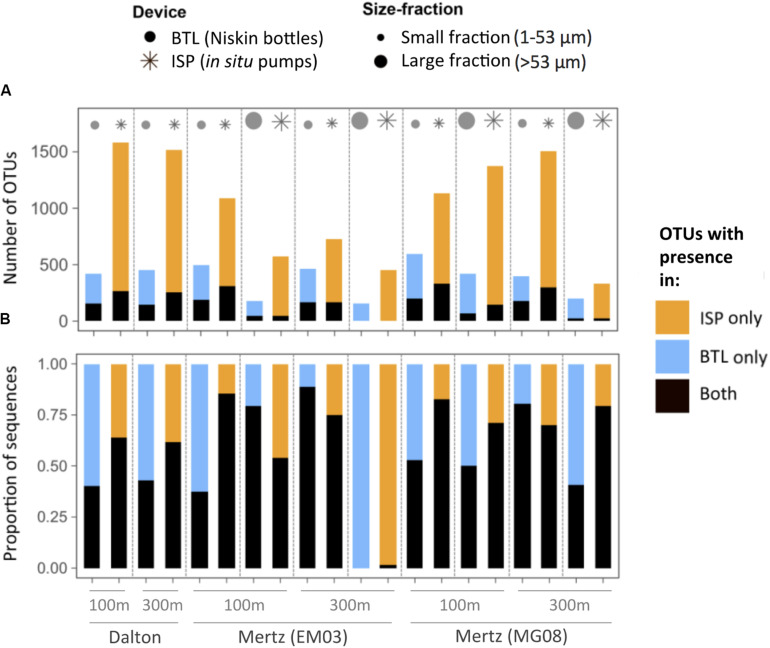
**(A)** Number of total OTUs detected in each sample, distinguishing between OTUs detected exclusively with *in situ* pumps (ISP, orange), with Niskin bottles (BTL, blue) or shared between both devices (black). **(B)** Contribution of these three groups of unique or shared OTUs to total community sequences. The depths and the stations sampled are indicated on the *X*-axis of panel **(B)**.

In order to assess if the device-driven structural differences involved changes in taxonomic richness and composition, for each particular community we classified the OTUs as only present in the BTL sample, only present in the ISP sample, or present in both types of samples (Figure 2A). Overall, we found that ISP-communities contained 1.5- to >3-fold more OTUs than BTL-communities at a similar sequencing depth (5,000 reads per sample). Remarkably, the majority of the OTUs (range 73–99%) found in a given ISP community were never detected in its BTL counterpart. Likewise, 60–97% of the OTUs in BTL communities were not recovered by the ISP. The “ISP-only” OTUs were mostly rare OTUs, representing in most cases a much lower proportion of the total number of sequences (14–46%) compared to the “BTL-only” OTUs, which accounted for a larger fraction of BTL-community sequences (11–62%) (Figure 2B). Only in one case (Mertz polynya, station EM03, 300 m depth) we found almost no overlap between the communities captured by the two methods (Figure 2B). In general, however, OTUs present in both ISP and BTL communities comprised a relatively large fraction of the sequences from each assemblage (range 39–88%, Figure 2B).

**FIGURE 3:**
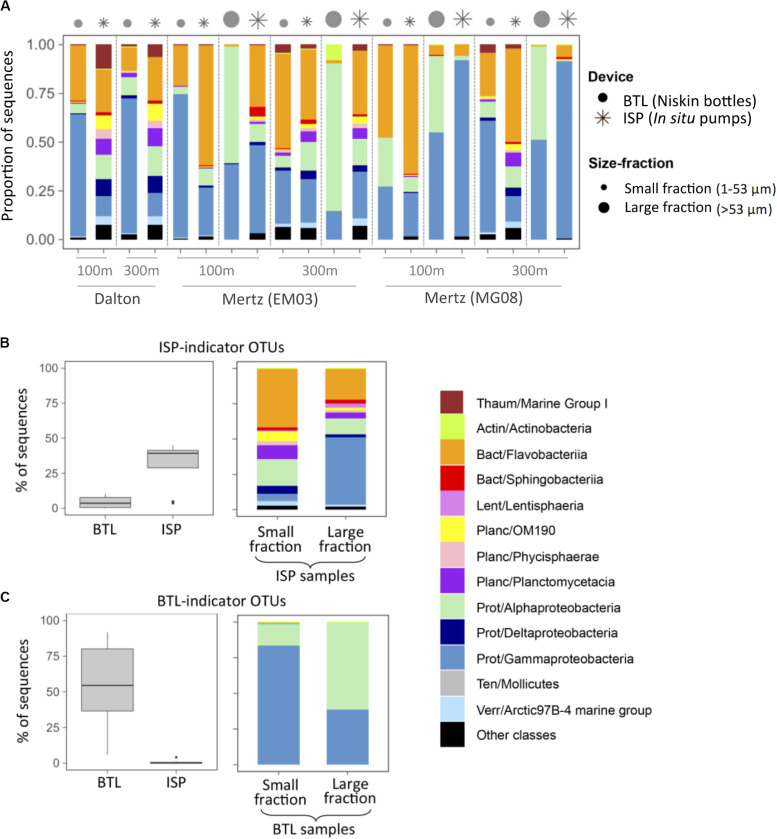
**(A)** Taxonomic composition of the communities sampled with either *in situ* pumps (ISP, asterisks) or Niskin bottles (BTL, dots). The size of the symbols indicates communities associated with small or large particles. The depths and the stations sampled are indicated on the *X*-axis of panel **(A)** (see Table 1). **(B,C)** Identification of ISP- or BTL-indicator OTUs (see section “Materials and Methods”). **(B)** Relative contribution (% of community sequences) and taxonomic composition of ISP-indicator OTUs. **(C)** Relative contribution (% of community sequences) and taxonomic composition of BTL-indicator OTUs. The classification was performed at the class level, and before each class, the phylum is indicated: Thaum, Thaumarchaeota; Actin, Actinobacteria; Bact, Bacteroidetes; Lent, Lentisphaerae; Planc, Planctomycetes; Prot, Proteobacteria; Ten, Tenericutes; Verr, Verrucomicrobia.

In most cases, ISP-communities harbored a higher diversity of groups, including OTUs belonging to Planctomycetes, Deltaproteobacteria, or Verrucomicrobia that were almost absent from BTL communities, which were mostly comprised by Alpha-, Gammaproteobacteria, or Flavobacteriia (Figure 3A and Supplementary Table S1). In order to explore whether there were specific taxa that were preferentially captured by each sampling strategy, we identified BTL- and ISP-indicator OTUs pooling together all BTL and all ISP samples, respectively. Out of the total 5,786 OTUs, we detected 123 ISP-indicators and 129 BTL-indicators (Figures 3B,C), which accounted for up to 49% of the ISP community sequences (Figure 3B) and up to 85% of the BTL community sequences (Figure 3C), respectively. These results suggest that there was preferential recovery depending on the sampling device, since BTL indicators comprised almost exclusively Alpha- and Gammaproteobacterial OTUs (Figure 3C), while OTUs belonging to Flavobacteriia, Sphingobacteriia, and several Planctomycetes groups were exclusively detected among ISP indicators (Figure 3B).

## Discussion

Since the advent of DNA sequencing technologies, there has been a rising number of studies on the ecology and diversity of prokaryotic communities associated to marine sinking particles (DeLong et al., 1993; Boeuf et al., 2019). However, there are still methodological considerations that limit our understanding of the microbial ecology of particles. For example, there is no clear consensus on the filter pore size to use to effectively separate particles of different kinds (see references in Mestre, 2017), known to be colonized by largely different microbial communities (Mestre et al., 2018; Duret et al., 2019). Although both *in situ* pumps and filtering of water collected with Niskin bottles have been used to sample particle-attached microbial communities (e.g., Lincoln et al., 2014; Mestre et al., 2018) there has been no attempt to compare whether these different methods yield a similar picture of particle-attached microbial assemblages, even though different sampling devices are known to collect chemically different particles (Liu et al., 2005, 2009; Puigcorbé et al., 2015; Twining et al., 2015). Here we provide the first comparison of the impact of two types of sampling devices, oceanographic bottles and *in situ* pumps, on the particle-attached microbial communities. Even though these two methods should capture essentially the same particles as they are not intended to preferentially collect sinking or suspended material, our results show that the microbial communities recovered are largely different.

Regardless of the method used, the taxonomic structure of the studied microbial communities differed strongly depending on the particle size, in agreement with previous investigations indicating that particles of different sizes are colonized by distinct microbial communities (e.g., Mestre et al., 2018). However, the differences due to the sampling device were larger than those due to the different sampling stations and depths. ISP and BTL-derived communities differed in terms of overall taxonomic structure, number of OTUs and identity of the taxa recovered, with ISP generally recovering more OTUs and a higher diversity of taxonomic groups than the BTL sampling. Hereafter we will discuss the potential causes underlying such differences, which include the use of different pore size filters, filter clogging, microbial community turnover during sample processing, or other sampling issues.

For practical reasons, size fractionation and sampled volumes between both sampling devices could not be identical. The filters used for collecting the small size-fractions were slightly different between the BTL and the ISP samples (Polycarbonate 0.8 μm vs. QMA 1 μm), which might have contributed to some of the differences observed in the sampled communities. However, the same type of 53 μm screen was used for the large size-fractions in both devices, and this size-fraction was precisely the one showing the greatest device-driven differences. This suggests that the difference in the pore size and filter type in small size fraction was not the main reason explaining the device-driven changes in microbial composition.

Taxonomic differences in particle-attached prokaryotes have sometimes been related to differences in the filtered volume. For example, taxonomic richness in prokaryotic free-living communities (0.22–1.6 μm) was found to decrease with increasing filtered volume (0.1–5 L), whereas it generally increased in the attached (>1.6 μm) fraction (Padilla et al., 2015). The authors claimed that this was due to progressive filter clogging (i.e., small cells being increasingly retained in the large fraction). Our ISP samples filtered on average four times more volume per filter area than BTL (see section “Materials and Methods”), which might have resulted in a more intense clogging of ISP than BTL filters. Based on the results of Padilla et al. (2015), clogging of ISP filters should have caused higher richness in ISP than in BTL samples in the large-size fractions, but the opposite pattern in the small size-fractions. As we recovered much higher taxonomic diversity of taxa in both size fractions in ISP samples, clogging might have not be the main reason explaining the differences between devices. Besides, we did not observe visual signs of clogging in any of the two fractions. As an alternative explanation, also suggested by Padilla et al. (2015), small volumes might fail to capture a fraction of the particles due to microscale patchiness in particle distribution (Long and Azam, 2001). Hence, the larger amount of water filtered by the ISP could be much more efficient at recovering certain types of rarer particles and their associated communities. Should this be the case, ISP would clearly be advantageous for the characterization of particle-attached communities with respect to Niskin bottles. Although it could be tested whether filtering larger volumes of water collected with Niskin bottles would lead to a more representative characterization of the particle-attached bacterial diversity, there are generally inherent practical difficulties in doing so given the limitations in water budgets and filtration times during oceanographic cruises.

Another potential bias could be due to microbial population turnover within the sample that may occur between particle collection and sample processing, as ISP samples are stored more quickly and are less manipulated than BTL samples; the ISP samples were collected during an average of 2 h pumping time, and in less than 1 h the filters were cut or rinsed and stored at −80°C. On the contrary, the BTL samples were collected during a CTD deployment (∼2 h), they then remained at low temperature (4°C) until filtration a few hours later, which took place at room temperature (∼20°C). Such a larger processing time between sample collection and final storage of the filters at −80°C could have allowed the growth of some opportunistic prokaryotic groups. The lower taxonomic richness observed in BTL communities actually points to the growth and dominance of a few taxa, and we observed higher relevance of some typical copiotrophic groups in the BTL samples, where Alteromonadales and Vibrionales were highly enriched (Supplementary Table S1). However, and as part of the larger study in which these results were obtained, we also sampled other stations and water depths with the ISP in which up to 80% of the sequences belonged to these bacterial groups (details not shown), which would suggest that their dominance is not solely the result of Niskin enclosure.

Interestingly, the sampling method seemed to be more relevant for communities associated to the large than to the small particles, as assemblages from large particles showed larger differences in taxonomic structure depending on the device used. When sampling with BTL, fast sinking particles, which differ in community composition from suspended particles (Duret et al., 2019) could accumulate at the bottom of the Niskin even during transit of the rosette to the surface and be undersampled, especially when not all content of the bottle is filtered (Suter et al., 2017). In addition, we observed that the large differences indicated by the NMDS between the ISP large size-fractions were not reflected by their BTL counterparts, which clustered more closely together in a single group. Similarly, the NMDS ordination patterns of the small size-fraction communities from the ISP and BTL communities did not resemble each other. This suggests that different sampling methods will affect not only the taxonomic composition and richness, but also the beta-diversity patterns (i.e., the observed differences between sites or stations) and hence, their links with environmental or spatio-temporal gradients. These discrepancies could be due to the previously mentioned growth of copiotrophs in oceanographic bottles, explaining the observed homogenization of BTL communities, or could be caused by fragmentation of large aggregates and loss of diversity during ISP filtration (Bishop et al., 2012; Maiti et al., 2012). The sampling device could also have impacted the observed taxonomic differences between small and large size-fractions. It could be hypothesized that large particles can be destroyed when collected with the BTL and converted into smaller particles because of the physical filtration with a peristaltic pump. This would imply smaller differences between large and small particles collected with the BTL than with the ISP. However, direct comparison of the Bray–Curtis dissimilarities (Table 2) indicate that in BTL samples there were larger or similar taxonomic differences between both particle sizes as compared to ISP samples, thus discarding this hypothesis. In any case, these contrasting patterns have important implications for our capacity to understand the ecological drivers of particle-attached communities.

Interestingly, despite the higher taxonomic richness recovered with ISP, we also detected a significant number of OTUs exclusive of BTL-samples that were not detected with ISP. We thus believe that the most likely explanation for the observed differences is that the two devices are capturing different types of particles. Indeed, other studies have unveiled differences in the chemical composition of particles collected by either BTL or ISP (Liu et al., 2005, 2009; Twining et al., 2015), which were attributed to sampling designs, undersampling of certain particle types, or loss of labile compounds. Similarly, the two studies that compared free-living assemblages (>0.22 or 0.44 μm) sampled with bottles or *in situ* filtration devices reported taxonomic differences (Torres-Beltrán et al., 2019; Wang et al., 2019) and suggested that *in situ* sampling could overcome biases linked to bottle sampling and provide a more accurate representation of the actual microbial structure. Our results agree with this interpretation and suggest that ISP generally capture a higher diversity of particle-attached microbes. For example, groups like Bacteroidetes, Planctomycetes, and Deltaproteobacteria, which were recovered preferentially with ISP, have shown a consistent preference for a particle-attached lifestyle across the global mesopelagic and bathypelagic ocean (Salazar et al., 2015; Mestre et al., 2018). While it is true that we cannot discard that some of the differences are caused by the differences in the amount of volume filtered, the higher diversity recovered by the ISP actually reflects a higher efficiency of this sampling device to capture the complexity of prokaryotic communities associated to particles in the ocean. It should be noted, however, that ISP also have potential biases, such as the rupture of fragile aggregates due to cross filter pressure, or the loss of some of the large particles collected with a mesh, due to washout when recovering the pumps (Bishop et al., 2012). In particular, the type of filter holders used in our study had been shown to lose large biogenic particles (Bishop et al., 2012), so it is possible that the differences in taxonomic richness between ISP and BTL large size fractions might be even larger than what we show here. We tried to minimize these issues by filtering at a flow rate of maximum 3.8 L min^–1^ [the limit to preserve sample integrity is thought to be 6 L min^–1^ (Maiti et al., 2012)], cared for a smooth device recovery, and drained the excess of water once the pumps were on deck. In any case, there is still a clear need to explore in detail the reasons behind the observed differences before we can unequivocally determine which sampling method is the most suitable for the characterization of particle-attached prokaryotic assemblages.

## Conclusion

Our results suggest that sampling marine particles with *in situ* pumps or oceanographic bottles provides a significantly different view of the particle-attached microbial communities recovered, calling for caution when studies using different methodologies are to be compared. We show that sampling with *in situ* pumps recovers more complex, diverse and heterogeneous particle-attached prokaryotic communities than sampling with Niskin bottles, and that some taxa are exclusively captured with one device or the other. To our knowledge, this is the first evidence of compositional differences in particle-attached assemblages caused by the sampling methods. Yet given that BTL and ISP are likely sampling different particle pools, combining devices may offer an opportunity for a more thorough understanding of the particle microbial ecology and its implications in the assessment of the carbon sequestration in the oceans.

## Data Availability Statement

The raw sequence data have been deposited in the Figshare data repository, together with the non-rarefied OTU table, the taxonomy table and the environmental data used in this study, 10.6084/m9.figshare.12333107.

## Author Contributions

VP, JG, and PM participated in the design of the sampling scheme. VP performed the sampling and sample processing. CR-G, VP, and JG conducted the data analysis. VP and CR-G compiled the needed data, analyzed the data, and wrote this manuscript. All authors made relevant comments and editions to the text.

## Conflict of Interest

The authors declare that the research was conducted in the absence of any commercial or financial relationships that could be construed as a potential conflict of interest.

## Footnotes

1 www.geotraces.org
