# Supplementary material for: Sampling Device-Dependence of Prokaryotic Community Structure on Marine Particles: Higher Diversity Recovered by in situ Pumps Than by Oceanographic Bottles
Source: Front Microbiol. 2020 Jul 15;11:1645. doi: 10.3389/fmicb.2020.01645 (PMC7373737; doi:10.3389/fmicb.2020.01645)
Supplement: Supplementary file 1 [file Image_1.pdf]

Supplementary Material

Supplementary Figure 1

Prior to assessing potential differences between sampling methods we evaluated the impact that subsampling of the ISP samples could have. Replicates were obtained for all the 1-53 µm samples and, when there was enough material, replicates were also obtained for the >53 µm samples. Results indicated that ISP replicates showed high similarities, discarding a bias due to subsampling: Bray-Curtis dissimilarities between replicates of a sample were much smaller (0.301±0.030) than between different samples (0.718 ± 0.007; Fig. S1).

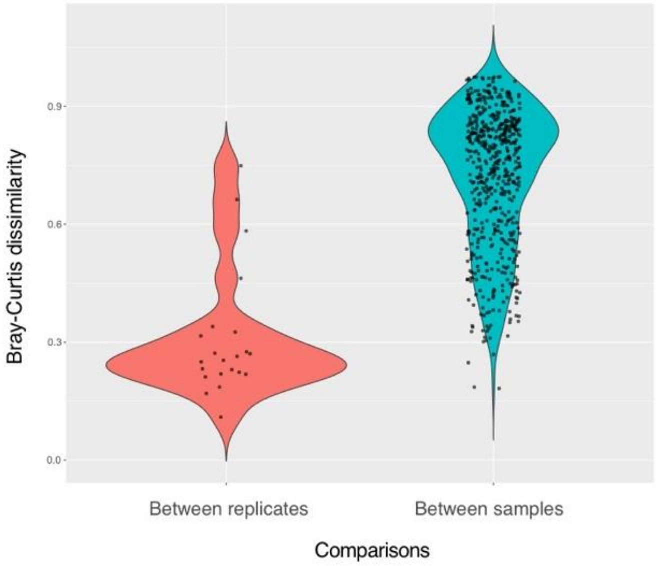

Supplementary Figure 1: Violin diagram representing the Bray-Curtis community dissimilarities between subsample replicates (red) and between samples (green)
